# CRISPR-MVLST subtyping of *Salmonella enterica* subsp. *enterica* serovars Typhimurium and Heidelberg and application in identifying outbreak isolates

**DOI:** 10.1186/1471-2180-13-254

**Published:** 2013-11-12

**Authors:** Nikki Shariat, Carol H Sandt, Michael J DiMarzio, Rodolphe Barrangou, Edward G Dudley

**Affiliations:** 1 Department of Food Science, The Pennsylvania State University, University Park, PA 16802, USA; 2 The Pennsylvania Department of Health, Bureau of Laboratories, Division of Clinical Microbiology, Exton, PA 19341, USA; 3 Current address: Department of Food, Bioprocessing and Nutrition Sciences, North Carolina State University, Raleigh, NC 27695, USA

**Keywords:** CRISPR subtyping, CRISPR-MVLST, Molecular subtyping, *Salmonella* Heidelberg, *Salmonella* Typhimurium

## Abstract

**Background:**

*Salmonella enterica* subsp. *enterica* serovars Typhimurium (*S*. Typhimurium) and Heidelberg (*S.* Heidelberg) are major causes of foodborne salmonellosis, accounting for a fifth of all annual salmonellosis cases in the United States. Rapid, efficient and accurate methods for identification are required for routine surveillance and to track specific strains during outbreaks. We used Pulsed-field Gel Electrophoresis (PFGE) and a recently developed molecular subtyping approach termed CRISPR-MVLST that exploits the hypervariable nature of virulence genes and Clustered Regularly Interspaced Short Palindromic Repeats (CRISPRs) to subtype clinical *S*. Typhimurium and *S*. Heidelberg isolates.

**Results:**

We analyzed a broad set of 175 *S.* Heidelberg and *S.* Typhimurium isolates collected over a five-year period. We identified 21 Heidelberg Sequence Types (HSTs) and 37 Typhimurium STs (TSTs) that were represented by 27 and 45 PFGE pulsotypes, respectively, and determined the discriminatory power of each method.

**Conclusions:**

For *S.* Heidelberg, our data shows that combined typing by both CRISPR-MVLST and PFGE provided a discriminatory power of 0.9213. Importantly, CRISPR-MVLST was able to separate common PFGE patterns such as JF6X01.0022 into distinct STs, thus providing significantly greater discriminatory power. Conversely, we show that subtyping by either CRISPR-MVLST or PFGE independently provides a sufficient discriminatory power (0.9345 and 0.9456, respectively) for *S.* Typhimurium. Additionally, using isolates from two *S.* Typhimurium outbreaks, we demonstrate that CRISPR-MVLST provides excellent epidemiologic concordance.

## Background

Non-typhoidal *Salmonella* are one of the leading causes of bacterial foodborne disease in the United States, accounting for over a million human cases each year [1]. Salmonellosis symptoms include diarrhea, fever and abdominal cramps that occur 12 to 72 hours after infection. Annually, *Salmonella* is responsible for an estimated 20,000 hospitalizations and nearly 400 deaths in the United States, with a financial burden of approximately $3.3 – 4.4 billion [2,3]. Most infections are transmitted via ingestion of contaminated food and, unlike trends with other bacterial foodborne pathogens, the annual incidence rate of salmonellosis has not significantly declined over the past decade. Since 2006, nearly a fifth of all salmonellosis cases in the United States were caused by *Salmonella enterica* subsp. *enterica* serovars Typhimurium (*S.* Typhimurium) and Heidelberg (*S.* Heidelberg) [4]. According to the Centers for Disease Control and Prevention, there have already been two outbreaks in 2013 where *S.* Typhimurium and *S.* Heidelberg were responsible [5,6].

To limit and reduce the scope of a *Salmonella* outbreak, an efficient and robust surveillance system is vital. During epidemiological investigations *Salmonella* isolates are serotyped and concurrently subtyped to classify isolates to the strain level. An ideal subtyping method has a high discriminatory power (i.e. can separate all unrelated strains) but is not so discriminatory that it inadvertently separates isolates that are part of the same outbreak (i.e. possesses high epidemiologic concordance). There are several molecular-based subtyping approaches that have been developed, including pulsed-field gel electrophoresis (PFGE) [7], amplified fragment length polymorphism (AFLP) [8-10], multiple-locus variable-number tandem-repeat analysis (MLVA) [11-17], multiple amplification of prophage locus typing (MAPLT) [13,18] and, most recently, a multiplex DNA suspension array [19]. PFGE was adapted to *Salmonella* in the 1990s and generally provides a high discriminatory power for subtyping most *Salmonella* serovars, though it certainly does not provide equal sensitivity across all serovars [20]. Despite being labor-intensive and time-consuming, conventional serotyping and concurrent PFGE fingerprinting is still considered the gold standard for *Salmonella* subtyping and is widely used by public health surveillance laboratories [21-23]. Although PFGE data are uploaded to PulseNet USA (http://www.cdc.gov/pulsenet), the national electronic network for food disease surveillance that is coordinated by the CDC, inter-laboratory comparisons of PFGE fingerprints can be ambiguous.

There are several different PFGE patterns, or pulsotypes, though most often a limited number of common patterns are associated with the majority of isolates within a given serovar. Two recent *S.* Typhimurium and *S.* Heidelberg foodborne outbreaks in the United States involved contaminated cantaloupe melons (*S.* Typhimurium, 2012; 228 reported illnesses) [24] and broiled chicken livers (*S.* Heidelberg, 2011; 190 reported illnesses) [25]. In both cases, the individual *Xba*I PFGE patterns associated with each strain were fairly common: for *S.* Typhimurium, the associated PFGE pattern is typically seen in 10–15 cases per month [24] and for *S.* Heidelberg, the pattern occurs even more frequently, 30–40 cases per month [25]. Consequently, identification of the outbreak strains was particularly difficult and to more accurately identify isolates that were part of the *S.* Typhimurium cantaloupe outbreak, these isolates were also analyzed by MVLA to define the outbreak strain. Additionally, another *S.* Heidelberg outbreak in 2011, linked to ground turkey, involved isolates with two similar but distinctly different PFGE patterns, thus showing reduced epidemiologic concordance by this subtyping method [26]. This last example may indicate evolutionary relatedness between the two sets of isolates which, unlike some methods, PFGE cannot really provide.

The recent outbreak cases described above highlight the need for additional subtyping approaches for *Salmonella* that can be used instead of, or as a complement to PFGE for routine disease surveillance and outbreak tracking. Clustered Regularly Interspaced Short Palindromic Repeats (CRISPRs) are found in ~50% of all bacterial species, including *Salmonella*[27]. CRISPR elements comprise several unique short sequences, called spacers, which are interspaced by conserved direct repeats. In some bacteria, homology between a spacer and a complementary target nucleic acid results in degradation of the target by sequence-specific endonucleases, providing protection from exogenous bacteriophage or plasmid DNA [reviewed in [28]. Due to both acquisition and loss of these spacer elements, CRISPRs represent arguably the most rapidly evolving prokaryotic loci [29-31].

**Figure 1:**
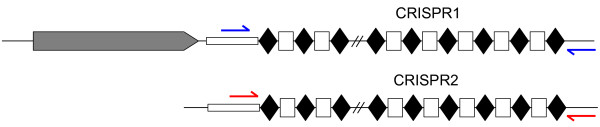
***Salmonella *CRISPR loci.***Salmonella* have two CRISPR loci, CRISPR1 and CRISPR2 comprised of direct repeats of 29 nucleotides (black diamonds) separated by spacers (empty rectangles). There is an A-T rich leader sequence upstream of each locus (shaded rectangle) and the *C*RISPR-*as*sociated genes (*cas*) are upstream of the CRISPR1 locus (grey boxed arrow). Primers used for amplification are shown in blue and red for CRISPR1 and CRISPR2, respectively.

Sequence analysis of CRISPR loci has been used to subtype clinical isolates of *Salmonella*[32-34], *Escherichia coli*[35,36], group A *Streptococcus*[37] and *Campylobacter* species [38]. *Salmonella* contains two of these non-coding loci, which are comprised of direct repeats of 29 nucleotides separated by spacers of 32 nucleotides (Figure 1). Generally, CRISPR polymorphisms between *Salmonella* strains are due to deletion or repetition of one or more spacers, termed ‘spacer microevolution’ [32-34,39,40]. An extensive investigation of 738 isolates, representing several different serovars, showed that polymorphisms within the CRISPR loci correlate highly with serovar, with isolates from individual serovars bearing distinct CRISPR patterns [32].

We recently developed a sequence-based subtyping assay (multi-virulence locus sequence typing; MVLST) for *Salmonella* that involves the sequencing of two virulence genes, *fimH1* (*fimH*) and *sseL*, in addition to CRISPR sequencing [33]. Preliminary studies showed that this approach, termed CRISPR-MVLST, provided better discrimination than either CRISPR or MVLST alone and, importantly, exhibited strong epidemiologic concordance among eight out of nine of the most common illness-causing *Salmonella enterica* serovars [33], including both *S.* Heidelberg and *S.* Typhimurium outbreak strains. Subsequently, among a large number of clinical isolates of the highly clonal *S.* Enteritidis, a combination of CRISPR-MVLST and PFGE was required to provide a sufficient discriminatory power [34]. Among a large set of *S.* Newport clinical isolates, CRISPR-MVLST provides similar discrimination to PFGE [41].

To further determine the functionality of this new subtyping approach, we investigated the discriminatory power of both CRISPR-MVLST and PFGE among a larger and unbiased collection of clinical S. Typhimurium and *S.* Heidelberg isolates that were collected over a five year period. We show here that a combination of both CRISPR-MVLST and PFGE is required to achieve an appropriate discriminatory power for *S.* Heidelberg. For *S.* Typhimurium, both subtyping methods independently provide a discriminatory power >0.94. Importantly, as one of the first applications of CRISPR-MVLST to analyze isolates that were part of an outbreak, we were able to cluster two different *S.* Typhimurium outbreak strains.

## Results

### Results of CRISPR-MVLST

**Figure 2:**
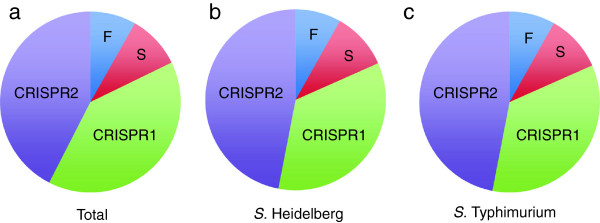
**Contribution of allele number for each marker.** Pie charts showing the combined total number of different alleles identified at all four loci. The contribution of each marker to this total is shown for **a)** combined all alleles from both *S.* Heidelberg and *S.* Typhimurium, **b)***S.* Heidelberg and **c)***S.* Typhimurium. F – *fimH*; S – *sseL*.

To more accurately determine the discriminatory power of CRISPR-MVLST and PFGE for *S.* Heidelberg and *S.* Typhimurium, we subtyped 89 and 86 isolates, respectively, that were obtained from the Pennsylvania Department of Health (Table 1). Among the 175 total isolates analyzed, we identified 29 CRISPR1 alleles, 31 CRISPR2 alleles, 6 *fimH* alleles and 7 *sseL* alleles (Table 2). Of these, we found 27, 30, 2 and 4 alleles, respectively, that were novel and not seen in our previous data sets [33]. In total, these alleles defined 58 novel sequence types among the two serovars (Tables 3 and 4). The overwhelming sequence-type diversity among both of these prevalent serovars is provided by genetic variability in the CRISPR loci, rather than in either *fimH* or *sseL* (Figure 2). We found that 88/89 *S.* Heidelberg isolates had *fimH* allele 7 and in *S.* Typhimurium there were two predominant *fimH* alleles, allele 6 (52/86 isolates) and allele 8 (28/86 isolates). Similarly, in *S.* Heidelberg, 88/89 isolates bore *sseL* allele 19 and in *S.* Typhimurium, 73/86 isolates had *sseL* allele 15. The polymorphisms between different *sseL* or *fimH* alleles arise from the presence of SNPs with the exception of allele 63 that has a single base insertion. No alleles for any of the four markers were shared among the two different serovars, consistent with previously published studies [32-34].

**Table 1:** **List of 175 *S. *** **Heidelberg and *S. *** **Typhimurium isolates from the Pennsylvania Department of Health that were analyzed in this study**

**Isolate**	**Sequence type**	**PFGE pattern**	**PA region**	**Isolation date**
***S. Heidelberg***				
06E00444	HST 7	JF6X01.0022	SE	Mar-06
06E00726	HST 7	JF6X01.0022	SE	Jun-06
06E01437	HST 7	JF6X01.0022	SE	Aug-06
07E00466	HST 7	JF6X01.0022	SE	Apr-07
07E00768	HST 7	JF6X01.0022	NC	May-07
07E01405	HST 7	JF6X01.0022	SE	Aug-07
07E01505	HST 7	JF6X01.0022	SE	Aug-07
08E00753	HST 7	JF6X01.0022	NE	Jun-08
08E01373	HST 7	JF6X01.0022	SE	Aug-08
09E00637	HST 7	JF6X01.0022	SE	Mar-09
09E00701	HST 7	JF6X01.0022	SE	Mar-09
09E00750	HST 7	JF6X01.0022	SE	Apr-09
09E00782	HST 7	JF6X01.0022	SE	Apr-09
09E01149	HST 7	JF6X01.0022	SE	May-09
09E01511	HST 7	JF6X01.0022	SE	Jun-09
M09019838001A	HST 7	JF6X01.0022	SE	Aug-09
M10003150001A	HST 7	JF6X01.0022	SE	Jan-10
M10014816001A	HST 7	JF6X01.0022	SE	Jun-10
M10016406001A	HST 7	JF6X01.0022	SE	Jul-10
M10022189001A	HST 7	JF6X01.0022	SE	Sep-10
M11012103001A	HST 7	JF6X01.0022	SW	Apr-11
M11017212001A	HST 7	JF6X01.0022	SE	Jul-11
M11021620001A	HST 7	JF6X01.0022	SW	Aug-11
06E00846	HST 7	JF6X01.0032	SW	Jun-06
08E00963	HST 7	JF6X01.0033	SW	Jul-08
08E01089	HST 7	JF6X01.0033	SE	Jul-08
07E01378	HST 7	JF6X01.0034	SW	Jul-07
08E00470	HST 7	JF6X01.0034	NE	May-08
08E00508	HST 7	JF6X01.0034	NE	May-08
M10000626001A	HST 7	JF6X01.0034	SW	Dec-09
07E00964	HST 7	JF6X01.0042	NW	Jun-07
M11025202001A	HST 7	JF6X01.0042	SC	Oct-11
M11027881001A	HST 7	JF6X01.0042	NE	Nov-11
07E01870	HST 7	JF6X01.0045	SC	Sep-07
M09021251001A	HST 7	JF6X01.0051	SE	Sep-09
09E00927	HST 7	JF6X01.0058	SE	May-09
08E00342	HST 7	JF6X01.0080	SE	Mar-08
M11018110001A	HST 7	JF6X01.0087	NW	Jul-11
06E00558	HST 7	JF6X01.0122	NW	
07E00680	HST 7	JF6X01.0122	SW	May-07
07E02336	HST 7	JF6X01.0161	SW	Nov-07
07E02139	HST 7	JF6X01.0167	SW	Oct-07
M09033280001A	HST 7	JF6X01.0221	SE	Dec-09
M10004098001A	HST 7	JF6X01.0246	SE	Feb-10
08E01461	HST 7	JF6X01.0324	SE	Aug-08
09E00128	HST 7	JF6X01.0324	SE	Jan-09
M09015668001A	HST 7	JF6X01.0326	SE	Jul-09
M10015955001A	HST 7	JF6X01.0581	SW	Jul-10
06E01523	HST 8	JF6X01.0051	SE	Sep-06
08E00143	HST 9	JF6X01.0022	NE	Feb-13
08E01679	HST 9	JF6X01.0022	SC	Sep-08
06E01915	HST 9	JF6X01.0022	SC	Oct-06
07E00349	HST 9	JF6X01.0022	SW	Feb-07
07E02366	HST 9	JF6X01.0022	NE	Dec-07
09E01408	HST 9	JF6X01.0022	SW	Jun-09
M10006052001A	HST 9	JF6X01.0022	SW	Mar-10
M10021328001A	HST 9	JF6X01.0022	SC	Sep-10
M11000821001A	HST 9	JF6X01.0041	NW	Jan-11
06E00519	HST 9	JF6X01.0052	NE	Apr-06
07E00933	HST 10	JF6X01.0051	SC	Jun-07
08E00107	HST 11	JF6X01.0085	NE	Jan-08
09E00226	HST 12	JF6X01.0022	SE	Jan-09
M10020282001A	HST 13	JF6X01.0034	NC	Sep-10
07E02483	HST 14	JF6X01.0022	SC	Dec-07
08E00103	HST 14	JF6X01.0022	SE	Jan-08
07E00451	HST 15	JF6X01.0049	SC	Mar-07
08E01904	HST 15	JF6X01.0049	SW	Sep-08
08E01911	HST 15	JF6X01.0049	SW	Oct-08
07E01400	HST 16	JF6X01.0270	SE	Jul-07
M10004892001A	HST 17	JF6X01.0041	SE	Mar-10
M11005464001A	HST 17	JF6X01.0041	SW	Feb-11
M11000267001A	HST 17	JF6X01.0500	NW	Dec-10
M09020244001A	HST 18	JF6X01.0321	SW	Aug-09
M09022904001A	HST 19	JF6X01.0022	NE	Sep-09
M11020321001A	HST 20	JF6X01.0042	SE	Aug-11
M10018092001A	HST 21	JF6X01.0033	SW	Aug-10
M11011342001A	HST 21	JF6X01.0058	SW	Apr-11
M11013202001A	HST 21	JF6X01.0058	SW	May-11
M11015845001A	HST 21	JF6X01.0058	SW	Jun-11
M11015850001A	HST 21	JF6X01.0058	SW	Jun-11
M11023722001A	HST 21	JF6X01.0058	SW	Sep-11
M11005685001A	HST 21	JF6X01.0582	SW	Feb-11
M10002453001A	HST 22	JF6X01.0032	SC	Jan-10
M09016444001A	HST 22	JF6X01.0033	NC	Jul-09
07E02184	HST 23	JF6X01.0042	SE	Oct-07
07E01907	HST 24	JF6X01.0058	SW	Sep-07
06E00416	HST 25	JF6X01.0172	NC	Mar-06
06E00661	HST 26	JF6X01.0022	SE	Jun-06
06E01299	HST 27	JF6X01.0022	SE	Aug-06
***S. Typhimurium***				
07E00002	TST 9	JPXX01.0177		Dec-06
07E02276	TST 9	JPXX01.0177		Nov-07
08E02063	TST 9	JPXX01.0177		Oct-08
09E00003	TST 9	JPXX01.0177		Dec-08
M09023403001A	TST 9	JPXX01.0177		Sep-09
07E01490	TST 10	JPXX01.0003		Aug-07
07E01769	TST 10	JPXX01.0003		Sep-07
07E02403	TST 10	JPXX01.0003		Dec-07
08E00363	TST 10	JPXX01.0003		Apr-08
09E00309	TST 10	JPXX01.0003		Jan-09
M10005050001A	TST 10	JPXX01.0003		Feb-10
M10010138001A	TST 10	JPXX01.0003		Apr-10
M10023515001A	TST 10	JPXX01.0003		Oct-10
07E00173	TST 10	JPXX01.0018		Jan-07
08E00006	TST 10	JPXX01.0018		Dec-07
M09017753001A	TST 10	JPXX01.0018		Jul-09
M10003149001A	TST 10	JPXX01.0018		Jan-10
M10006054001A	TST 10	JPXX01.0098		Mar-10
07E00658	TST 10	JPXX01.0256		Apr-07
08E00457	TST 10	JPXX01.1011		Apr-08
M10018865001A	TST 10	JPXX01.2731		Aug-10
07E00234	TST 11	JPXX01.0442		Feb-07
M10001003001A	TST 11	JPXX01.0442		Jan-10
07E00290	TST 12	JPXX01.0022		Feb-07
07E00436	TST 12	JPXX01.0146		Mar-07
M09028540001A	TST 12	JPXX01.0146		Oct-09
M10012000001A	TST 12	JPXX01.0146		May-10
M11018826001A	TST 12	JPXX01.0604		Jul-11
09E01310	TST 12	JPXX01.0925		May-09
08E02215	TST 12	JPXX01.1302		Nov-08
08E00255	TST 13	JPXX01.0001		Feb-08
M11021986001A	TST 13	JPXX01.0081		Aug-11
09E00084	TST 13	JPXX01.0111		Dec-08
07E00868	TST 13	JPXX01.0206		Jun-07
07E00568	TST 13	JPXX01.0642		Apr-07
07E00364	TST 13	JPXX01.1212		Jan-07
07E01042	TST 14	JPXX01.1393		Jun-07
07E01180	TST 15	JPXX01.0003		Jun-07
08E01211	TST 15	JPXX01.0003		Jul-08
M11004438001A	TST 15	JPXX01.0003		Jan-11
M11016520001A	TST 15	JPXX01.0070		Jun-11
07E01365	TST 16	JPXX01.0928		Jul-07
08E00877	TST 17	JPXX01.0006		Jun-08
08E01423	TST 17	JPXX01.0006		Aug-08
07E02063	TST 17	JPXX01.0146		Oct-07
M09025088001A	TST 17	JPXX01.0146		Oct-09
M11002975001A	TST 17	JPXX01.0146		Jan-11
08E01686	TST 17	JPXX01.0416		Sep-08
07E02348	TST 18	JPXX01.0018		Nov-07
08E00618	TST 19	JPXX01.0146		May-08
M10000110001A	TST 19	JPXX01.0146		Jan-10
M10010755001A	TST 19	JPXX01.0146		May-10
M11025544001A	TST 19	JPXX01.0146		Sep-11
08E00074	TST 19	JPXX01.0557		Jan-08
M11011894001A	TST 19	JPXX01.2900		Apr-11
M09018928001A	TST 20	JPXX01.0001		Aug-09
08E00162	TST 20	JPXX01.0014		Feb-08
09E00747	TST 20	JPXX01.0014		Apr-09
M11029619001A	TST 20	JPXX01.0014		Nov-11
M10026894001A	TST 20	JPXX01.0146		Nov-10
08E00998	TST 21	JPXX01.0604		Jul-08
08E02429	TST 22	JPXX01.1396		Dec-08
09E00422	TST 23	JPXX01.1255		Feb-09
09E00632	TST 24	JPXX01.1975		Mar-09
09E00904	TST 25	JPXX01.2016		Apr-09
M09014919001A	TST 26	JPXX01.0083		Jun-09
M09015997001A	TST 27	JPXX01.0416		Jul-09
M09020496001A	TST 28	JPXX01.0146		Aug-09
M09021700001A	TST 29	JPXX01.0552		Sep-09
M10014370001A	TST 30	JPXX01.0333		Jun-10
M10015309001A	TST 31	JPXX01.0003		Jun-10
M10016817001A	TST 32	JPXX01.0324		Jul-10
M10025067001A	TST 33	JPXX01.0359		Oct-10
M10028492001A	TST 34	JPXX01.0060		Dec-10
M11001607001A	TST 35	JPXX01.0359		Jan-11
M11009301001A	TST 36	JPXX01.1678		Mar-11
M11012744001A	TST 37	JPXX01.0013		May-11
M11015184001A	TST 38	JPXX01.1833		Jun-11
M11022803001A	TST 39	JPXX01.0146		Sep-11
M10007760001A	TST 40	JPXX01.2488		Apr-10
M11006620001A	TST 41	JPXX01.1314		Feb-11
M11024498001A	TST 42	JPXX01.0351		Oct-11
09E01078	TST 42	JPXX01.0781		May-09
07E00784	TST 56	JPXX01.0359		May-07
08E00321	TST 57	JPXX01.1301		Mar-08
M09031352001A	TST 58	JPXX01.0146		Nov-09

The data are shown in order of Sequence Type (HST or TST) and further sorted by PFGE pattern.

**Table 2:** Number of alleles identified for each of the four CRISPR-MVLST markers

**Serovar**	***fimH***	***sseL***	**CRISPR1**	**CRISPR2**
*S.* Heidelberg	0 (2)	1 (2)	12 (12)	7 (8)
*S.* Typhimurium	2 (4)	3 (5)	13 (15)	19 (19)
Total	2 (6)	4 (7)	25 (27)	26 (27)

The total number of alleles for each locus is listed in parentheses with the number of alleles that are new in this study, as compared to Liu et al. [33], shown to the left.

**Table 3:** **List of all *S. H*** **eidelberg CRISPR-MVLST *S*** **equence *T*** **ypes (HSTs) that were identified in this study**

**HST**	**Frequency**	**Allelic profile**			
		***fimH***	***sseL***	**CRISPR1**	**CRISPR2**
HST 7	*48*	17	19	167	32
HST 8	*1*	17	19	168	209
HST 9	*10*	17	19	167	209
HST 10	*1*	17	19	169	32
HST 11	*1*	17	19	170	32
HST 12	*1*	17	19	171	32
HST 13	*1*	18	19	167	32
HST 14	*2*	17	19	179	32
HST 15	*3*	17	19	167	212
HST 16	*1*	17	19	173	213
HST 17	*3*	17	19	172	32
HST 18	*1*	17	19	178	32
HST 19	*1*	17	67	174	209
HST 20	*1*	17	19	175	32
HST 21	*7*	17	19	167	211
HST 22	*2*	17	19	167	210
HST 23	*1*	17	19	177	32
HST 24	*1*	17	19	167	214
HST 25	*1*	17	19	176	32
HST 26	*1*	17	19	177	215
HST 27	*1*	17	19	167	215

The numbers represent the allelic identifier for the individual CRISPR-MVLST markers. The combination of four specific alleles defines a given HST. The frequency is the number of times a particular HST was observed among the 89 *S.* Heidelberg isolates analyzed. All HSTs identified here were new and not seen in previous studies.

**Table 4:** **List of all *S. T*** **yphiurium CRISPR-MVLST *S*** **equence *T*** **ypes (TSTs) that were identified in this study**

**TST**	**Frequency**	**Allelic profile**			
		***fimH***	***sseL***	**CRISPR1**	**CRISPR2**^**a**^
TST 9	*5*	6	15	129	159*
TST 10	*16*	8	15	11	160
TST 11	*2*	6	15	10	163*
TST 12	*7*	6	15	10	164*
TST 13	*6*	6	15	129	162
TST 14	*1*	6	15	129	165
TST 15	*4*	8	15	11	161
TST 16	*1*	8	61	11	160
TST 17	*6*	6	15	10	167*
TST 18	*1*	8	20	131	160
TST 19	*6*	6	62	10	164*
TST 20	*5*	49	15	129	162
TST 21	*1*	6	15	132	164*
TST 22	*1*	6	15	10	168*
TST 23	*1*	8	20	11	160
TST 24	*1*	6	15	133	167*
TST 25	*1*	50	20	134	169*
TST 26	*1*	6	15	10	170*
TST 27	*1*	6	15	10	171*
TST 28	*1*	6	15	10	172*
TST 29	*1*	8	62	11	160
TST 30	*1*	6	15	137	174
TST 31	*1*	8	15	11	175
TST 32	*1*	6	15	135	162
TST 33	*1*	6	15	138	177*
TST 34	*1*	8	15	139	161
TST 35	*1*	6	15	140	178*
TST 36	*1*	8	63	11	160
TST 37	*1*	6	15	141	162
TST 38	*1*	6	15	10	179*
TST 39	*1*	6	15	10	180*
TST 40	*1*	6	15	142	173*
TST 41	*1*	8	20	143	166
TST 42	*2*	6	15	10	181**
TST 56	*1*	6	15	130	173*
TST 57	*1*	6	15	10	205**
TST 58	*1*	6	15	136	164*
TST 59	-	6	62	10	207*
TST 60	-	6	15	166	208*

The numbers represent the allelic identifier for the individual CRISPR-MVLST markers. The combination of four specific alleles defines a given HST. The frequency is the number of times a particular TST was observed among the 86 *S.* Typhimurium isolates analyzed in the first study and does not include the frequency of TSTs that were seen in the outbreak study. All TSTs identified here were new and not seen in previous studies. ^a^Some CRISPR2 alleles required more than two sequencing primers to cover the whole length of the array. Alleles that required three primers are noted with * and the two isolates that required seven primers to sequence CRISPR2 are noted with **. The position of these primers is shown in Additional file 1.

### *S*. Heidelberg analysis and sequence type distribution

CRISPR-MVLST analysis of 89 *S*. Heidelberg clinical isolates (representing 27 unique PFGE patterns) resulted in 21 unique *S. H*eidelberg *S*equence *T*ypes (HSTs), HST 7 – HST 27 (Table 3). In total, we identified 12 CRISPR1 alleles, 8 CRISPR2 alleles, 2 *fimH* alleles and 2 *sseL* alleles (Table 2). As shown in Figure 2b, most of the allelic diversity comes from the CRISPR1 and CRISPR2 loci. All 12 CRISPR1 alleles and seven of the eight CRISPR2 alleles were new, compared to our previous studies [33]. We did not find any new *fimH* alleles in our dataset and only one of the two *sseL* alleles was new. The most frequent ST was HST7, occurring in 49/89 isolates (54%).

### Discriminatory power of CRISPR-MVLST and PFGE in *S.* Heidelberg isolates

**Figure 3:**
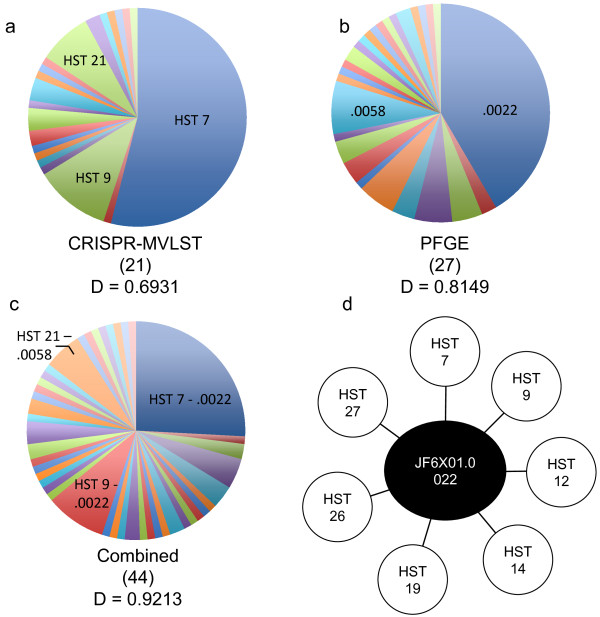
**Frequency of *S. *Heidelberg subtype prevalence generated by CRISPR-MVLST and PFGE.** Pie charts showing the number and frequency of distinct subtypes defined by **a)** CRISPR-MVLST, **b)** PFGE and **c)** the combination of CRISPR-MVLST and PFGE among 89 *S.* Heidelberg isolates. The most frequent subtypes for each method are indicated; .0022 and .0058 represent PFGE profiles JF6X01.0022 and JF6X01.0058, respectively. The number of distinct subtypes defined by each method is listed in parenthesis and the discriminatory power (D) is listed below. **d)** CRISPR-MVLST is able to separate the most common *S.* Heidelberg PFGE pattern JF6X01.0022 into 7 distinct sequence types.

The discriminatory power of CRISPR-MVLST among the *S.* Heidelberg isolates was calculated to be 0.6931 (Figure 3a). The discriminatory power provided by PFGE among the same isolates was 0.8149 (Figure 3b). Given these low values and insufficient discriminatory power (an ideal discriminatory power is >0.95) [42], we combined the two typing methods. This combination provided 44 unique groups with a more satisfactory discriminatory power of 0.9213 (Figure 3c), suggesting a 92% confidence in ability to separate unrelated isolates.

### Separation of common *S*. Heidelberg subtypes

Among the *S.* Heidelberg isolates analyzed, the most frequent PFGE pulsotype was JF6X01.0022 (42%). We were able to further subtype isolates with JF6X01.0022 pattern into 7 distinct HSTs - HST 7, 9, 12, 14, 19, 26 and 27 (Figure 3d). Among JF6X01.0022 isolates, the two most common HSTs were HST7 (62%) and HST9 (22%). JF6X01.0058 is also fairly common, occurring in 8% of isolates studied. With these isolates, we were able to further subtype them into 3 distinct HSTs – HST 7, 21 and 24, with HST21 being the most common (71%). Conversely, over half the isolates analyzed have HST 7 (54%), but by PFGE analysis, these are represented by 18 different PFGE patterns, the most frequent being JF6X01.0022 (48%). Collectively, this data highlights the strengths and weakness of each subtyping method.

### *S.* Typhimurium analysis and sequence type distribution

CRISPR-MVLST analysis of 86 *S*. Typhimurium clinical isolates (representing 45 unique PFGE patterns) resulted in the identification of 37 unique and novel *S. T*yphimurium *S*equence *T*ypes (TSTs), TST9 – TST41, and TST56 – TST58 (Table 4). This included 17 CRISPR1, 23 CRISPR2, 4 *fimH* and 5 *sseL* alleles (Table 2). Of these, the majority of CRISPR1 alleles were new (15/17 alleles) and all CRISPR2 alleles were new (23/23), as compared to our previous studies [33]. As with *S.* Heidelberg, the majority of unique sequence types were defined by polymorphisms in either or both of the CRISPR loci (Figure 2c).

### Discriminatory power of CRISPR-MVLST and PFGE in *S*. Typhimurium isolates

**Figure 4:**
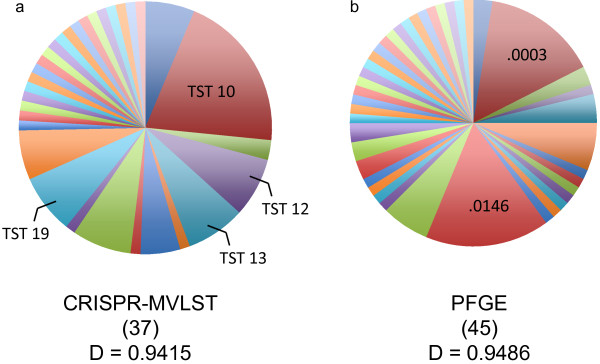
**Frequency of *S. *Typhimurium subtype prevalence generated by CRISPR-MVLST and PFGE.** Pie charts showing the number of distinct subtypes defined by **a)** CRISPR-MVLST and **b)** PFGE among 86 *S.* Typhimurium isolates. The most frequent TSTs or PFGE patterns observed are indicated. .0003 and .0146 represent PFGE profiles JPXX01.0003 and JPXX01.0146, respectively. The number of distinct subtypes defined by each method is listed in parenthesis and the discriminatory power (D) is listed below.

The discriminatory power of CRISPR-MVLST among the *S.* Typhimurium isolates was 0.9415 (Figure 4a). This means that there would be a 94% probability that two unrelated isolates could be separated using the CRISPR-MVLST scheme. Similarly, for PFGE, the discriminatory power among these isolates is 0.9486 (Figure 4b). These values suggest that either method can provide sufficient discrimination between outbreak and non-outbreak *S.* Typhimurium strains.

### Correlation between different TSTs and PFGE patterns

**Figure 5:**
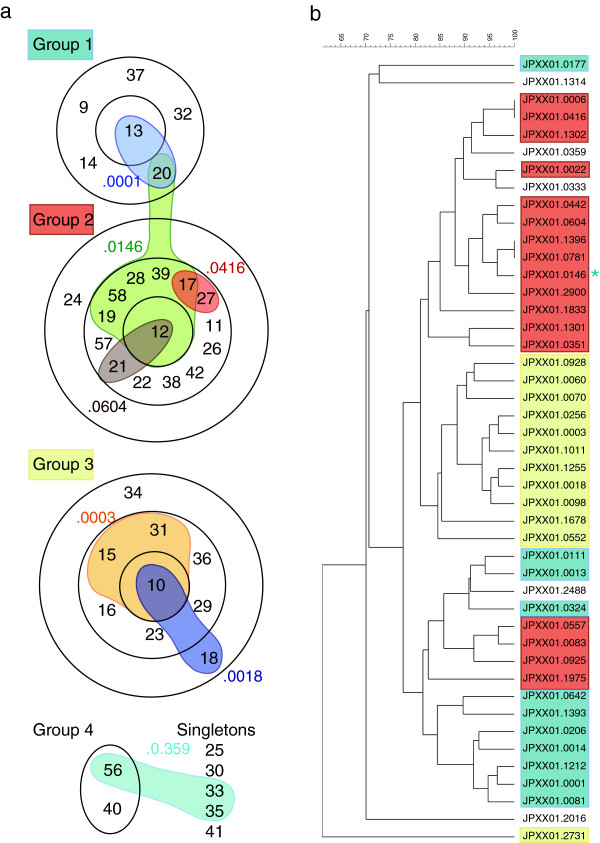
**Correlation of CRISPR-MVLST and PFGE. a)** BURST analysis of 37 TSTs identified in this study shows the relationship between different TSTs. Within a BURST group, the TSTs within one ring differ from TSTs in an adjacent ring at one of the four CRISPR-MVLST loci. TSTs that could not be assigned to a group are listed as singletons. Individual PFGE patterns that are found in isolates that have different TSTs are shown in color and the PFGE pulsotype is indicated as the numbers after JPXX01, i.e. JPXX01.0604 is shown as .0604. **b)** Dendrogram showing the levels of similarity between the 45 different PFGE patterns identified. All the PFGE patterns that are found in isolates with TSTs in Groups 1–3 are shaded in the corresponding color. The blue asterix represents TST 20, which is in Group 1.

We next wanted to investigate whether any correlation existed between TSTs and PFGE patterns. To accomplish this, we first determined the relationship among different TSTs. BURST analysis of all 37 TSTs generated four groups (Figure 5a). Of these, Groups 1–3 contain 6 – 15 TSTs. Group 4 consists of only two TSTs and BURST was unable to assign a core TST. There was also a collection of five singletons that BURST did not assign to a group. For Groups 1–3, each group comprises a core TST surrounded by TSTs that differ from the core by one allele. The number of rings in the group demonstrates the number of allele differences from the core. For example, in Group 1 TSTs 9, 37, 32, 20, and 14 each differ by one allele at one locus from the core TST, TST 13. For group 3, TST 10 is the core TST and TSTs 15, 31, 36, 29, 23 and 16 each differ from TST 10 at one locus. TST 34, in the outer ring differs from the TSTs in the middle ring at one locus and from the core at two loci.

To investigate whether there was any relationship between CRISPR-MVLST sequence type and PFGE patterns, we overlaid our PFGE data to identify isolates from different TSTs that have the same PFGE pattern. Figure 5a shows that there were seven PFGE pulsotypes that could be further separated into TSTs. In the majority of instances (5/7), identical PFGE patterns were found in isolates that had closely related TSTs such as JPXX01.0003 and JPXX01.0604 (TSTs 15, 31, 10 and TSTs 12 and 21, respectively).

Following this, we then generated a dendrogram using the Dice coefficient to determine the relationship between different PFGE pulsotypes. For clarity, we color-coded the PFGE patterns according to the BURST Group shown in Figure 5a. As can be seen in Figure 5b, closely related CRISPR-MVLST sequence types have similar PFGE patterns.

### CRISPR-MVLST analysis of *S*. Typhimurium outbreak isolates

Since CRISPR-MVLST and PFGE exhibit a similarly high discriminatory ability in *S.* Typhimurium, we wanted to investigate the utility of the former for separating outbreak isolates. We obtained 30 *S.* Typhimurium isolates from the Pennsylvania Department of Health (Table 5). Ten of these were isolates associated with an outbreak in 2004 with the cluster designation 0411PAJPX-1c. All affected persons were on a bus trip together, though the outbreak source was never identified. The remaining 20 isolates comprised 10 isolates that were linked to a 2009 live poultry outbreak (cluster 0905PAJPX-1) and 10 control isolates that were isolated in the same year but were not part of any classified outbreaks.

**Table 5:** **List of 30 *S. *** **Typhimurium isolates used in the outbreak study**

**Isolate**	**Sequence type**	**PFGE-pattern ( *Xba *** **I)**	**PFGE pattern ( *Bln *** **I)**	**Outbreak cluster**
04E02240	TST 59	JPXX01.0146	JPXA26.0172	0411PAJPX-1c
04E02241	TST 59	JPXX01.0146	**JPXA26.0294**	0411PAJPX-1c
04E02243	TST 59	JPXX01.0146	JPXA26.0172	0411PAJPX-1c
04E02295	TST 59	JPXX01.0146	JPXA26.0172	0411PAJPX-1c
04E02296	TST 59	JPXX01.0146	JPXA26.0172	0411PAJPX-1c
04E02297	TST 59	JPXX01.0146	JPXA26.0172	0411PAJPX-1c
04 F00368	TST 59	JPXX01.0146	JPXA26.0172	0411PAJPX-1c
04 F00376	TST 59	JPXX01.0146	JPXA26.0172	0411PAJPX-1c
04 F00381	TST 59	JPXX01.0146	JPXA26.0172	0411PAJPX-1c
04E02239	TST 59	**JPXX01.0279**	JPXA26.0172	0411PAJPX-1c
09E00857	TST 42	JPXX01.0302	JPXA26.0183	0905PAJPX-1
09E01235	TST 42	JPXX01.0302	JPXA26.0183	0905PAJPX-1
09E01308	TST 42	JPXX01.0302	JPXA26.0183	0905PAJPX-1
09E01333	TST 42	JPXX01.0302	JPXA26.0183	0905PAJPX-1
09E01424	TST 42	JPXX01.0302	JPXA26.0183	0905PAJPX-1
09E01666	TST 42	JPXX01.0302	JPXA26.0183	0905PAJPX-1
M09015209001A	TST 42	JPXX01.0302	JPXA26.0183	0905PAJPX-1
M09017319001A	TST 42	JPXX01.0302	JPXA26.0183	0905PAJPX-1
M09019457001A	TST 42	JPXX01.0302	JPXA26.0183	0905PAJPX-1
M09021164001A	TST 42	JPXX01.0302	JPXA26.0183	0905PAJPX-1
M09015294001A	**TST 42**	JPXX01.0047	**-**	**-**
M09019934001A	**TST 42**	JPXX01.0781	**-**	**-**
M09015723001A	TST 12	JPXX01.0604	JPXA26.0292	**-**
M09019606001A	TST 12	JPXX01.0604	JPXA26.0174	**-**
M09016911001A	TST 12	JPXX01.1214	**-**	**-**
09E00951	TST 13	JPXX01.0001	JPXA26.0530	**-**
M09019186001A	TST 13	JPXX01.0946	**-**	**-**
09E01471	TST 15	JPXX01.2095	**-**	**-**
M09016893001A	TST 19	JPXX01.0146	JPXA26.0291	**-**
M09017200001A	TST 60	JPXX01.0359	**-**	**-**

The 10 isolates without cluster information represent the sporadic, or non-outbreak related, isolates used as controls in the study.

CRISPR-MVLST was able to separate the 2004 isolates, with each isolate bearing the unique TST59 (Tables 4 and 5). These isolates were also analyzed by two-enzyme PFGE, using *Xba*I and *Bln*I. Though they had the same TST, two of the isolates, 04E02241 and 04E02239 had different PFGE patterns with *Bln*I or *Xba*I, respectively, and are indicated in bold in Table 5. This example shows that CRISPR-MVLST provides an epidemiologic concordance of 1 (E = 1.0) and for PFGE it is less than 1 (E < 1.0). Additionally, the *Xba*I PFGE pattern associated with this strain, JPXX01.0146, occurred fairly frequently in our initial data set; 12/86 isolates had this pulsotype and we were able to separate these into seven different TSTs.

For the 2009 outbreak isolates, CRISPR-MVLST correctly identified the 10 outbreak isolates (TST42) and these all have the same PFGE pattern, JPXX01.0302, thus for both subtyping methods E = 1.0. Two of the sporadic case control isolates were also TST42 (shown in bold in Table 5) but these had different PFGE pulsotypes from the outbreak strain, suggesting a lack of discrimination by CRISPR-MVLST in this instance. TST42 was seen in two isolates in the initial study of 86 *S.* Typhimurium isolates. All isolates within each outbreak were identified using CRISPR-MVLST, thus obtaining perfect epidemiological concordance with this subtyping method.

## Discussion

Foodborne illness caused by *Salmonella enterica* species, particularly by *S.* Typhimurium and *S.* Heidelberg, accounts for 18.5% of salmonellosis annually in the United States [4]. For accurate outbreak tracking and routine disease surveillance, it is critical that we employ rapid, efficient and robust subtyping methodologies. PFGE is the current gold standard for molecular subtyping of *Salmonella* and other methods include AFLP, MVLA and CRISPR-MVLST.

CRISPR sequence analysis is one of the cheaper and faster methods for *Salmonella* subtyping [22]. For the majority of isolates analyzed, CRISPR-MVLST could be completed in less than 24 hours, including DNA isolation and analysis. Additionally, by virtue of their nature, sequencing data are more robust and tractable; this type of data is unequivocal and, with regards to inter-laboratory or database use, is highly consistent. They also provide increased downstream utilities that involve analysis of sequence information, such as phylogenetic studies. This approach is also in line with other high-throughput subtyping approaches, including real-time CRISPR analysis [32] and whole genome sequence analysis [43-47]. Conversely, although protocols exist that allow PFGE to be completed in 24 hours, it can often take 1–3 days, requires skilled personnel, inter-laboratory data analysis can be challenging and the data have no utility beyond subtyping. Given the advancement of whole-genome sequencing technologies, typing methods based on these are in development [48]. While highly discriminatory, limitations to this approach that are not issues with either CRISPR-MVLST or PFGE include the time required for analysis and space required for data storage.

CRISPR spacer analysis alone has been used to analyze several different *Salmonella* serovars [32]. Fabre and colleagues showed that among 50 isolates of *S.* Typhimurium and its I,4, [5],12:i- variant, combined CRISPR1 and CRISPR2 sequence information is comparable to PFGE (D = 0.88 and 0.87, respectively). Both methods were more discriminatory than phage typing analysis of the same set of isolates. The same study also analyzed spacer content of *S.* Typhimurium and *S.* Enteritidis from 10 outbreaks and in all cases CRISPR sequences exhibited high epidemiologic concordance.

A preliminary investigation showed that addition of CRISPR spacer analysis to an MVLST scheme improves discrimination, beyond that provided by either approach independently, in eight out of nine of the most common illness-causing *Salmonella* serovars [33]. We wanted to extend our evaluation of CRISPR-MVLST utility among predominant and clinically relevant *Salmonella* serovars. To date we have tested and compared CRISPR-MVLST to PFGE on large numbers of *S.* Enteritidis [34], *S.* Newport [41]*S*, Heidelberg and *S*. Typhimurium isolates. Among the total 175 isolates analyzed here, we found significantly fewer alleles of *fimH* and *sseL*, compared to alleles of either CRISPR locus (Table 2; Figure 2). Given the reduced contribution of the virulence genes to defining STs, their addition may seem superfluous within this subtyping scheme. However, in this data set, *fimH* alleles define two STs, HST13 and TST20 and *sseL* alleles define five STs, TST16, TST19, TST23, TST29 and TST36. This further supports earlier findings showing that addition of MVLST to a CRISPR-based subtyping scheme increases discrimination in *S.* Enteritidis [34] as well as among a broad set of *Salmonella enterica* serovars [33].

Though the number of isolates for each serovar was similar, the number of STs within each serovar is surprisingly disparate: among 89 *S.* Heidelberg isolates we identified 21 HSTs and in 86 *S.* Typhimurium isolates, we identified 37 TSTs. This presumably reflects varied levels of clonality in different serovars. Independently of the number of STs defined for either serovar, the CRISPR loci are responsible for the vast majority of alleles: (*S.* Heidelberg – 83.3% and *S.* Typhimurium – 80%) (Figure 2). In *S.* Heidelberg, 50% of the different alleles identified were CRISPR1 alleles. Given that CRISPRs are of one of the more dynamic loci in bacteria [30,31], this finding is not unexpected.

Although PFGE was more discriminatory than CRISPR-MVLST among 89 *S.* Heidelberg isolates (D = 0.81 versus 0.69, respectively), a combination of both techniques provided an improved value of 0.92. This represents a 92% probability that two unrelated strains can be separated. JF6X01.0022 is the most common PFGE pattern in PulseNet for *S.* Heidelberg [49] and is seen 30–40 times a month by the CDC. In our data set, 42% of the isolates have the JF6X01.0022 pattern and using CRISPR-MVLST, we were able to further separate these into seven distinct CRISPR-MVLST types (Figure 3b and d). Given the frequency at which this PFGE pattern occurs nationally, not all isolates that have this pattern may be associated with a specific outbreak, further enhancing the utility of CRISPR-MVLST as a complement to PFGE analysis. Collectively, these findings in *S.* Heidelberg show that the JF6X01.0022 pattern is analogous to the JEGX01.0004 pattern in *S.* Enteritidis, where the latter was observed in 51% of isolates analyzed and was separated into 12 distinct STs [34]. A proposed improvement for discrimination in *S.* Heidelberg and *S.* Enteritidis by PFGE is to increase the number of enzymes used for PFGE analysis [50,51], though the concurrent use of PFGE and CRISPR-MVLST would be much more efficient than this approach.

Regarding *S.* Heidelberg, our data are similar to that observed in a broad set of *S.* Enteritidis isolates [34]: both serovars exhibit fewer number of STs identified and both require combining CRISPR-MVLST and PFGE to obtain a sufficient discriminatory power. This presumably reflects similar levels of clonality in *S.* Heidelberg and *S.* Enteritidis as compared to more heterogenous serovars such as *S.* Typhimurium where we observed many more STs present within a similar number of isolates examined.

Our data show that in *S.* Typhimurium, the discrimination provided by either PFGE or CRISPR-MVLST is similar (0.9486 versus 0.9415, respectively). When CRISPR-MVLST was applied to outbreak isolates, we were able to correctly identify the 20 isolates representing the two outbreaks, showing an extremely good epidemiologic concordance with this typing method. The epidemiologic concordance was better by CRISPR-MVLST than PFGE in identifying isolates from the 2004 bus trip outbreak and both methods had equal epidemiological concordance for the 2009 live poultry outbreak. Regarding the 2004 outbreak, the majority of isolates had the JPXX01.0146 pulsotype. In our initial study, this pulsotype was seen frequently, 16% of all isolates analyzed, and the 14 isolates with this pattern could also be represented by 7 distinct TSTs. Conversely, all isolates from this outbreak have TST59, which is unique and not seen in our initial data set showing that in this instance, CRISPR-MVLST may be a better subtyping approach. In analyzing the 2009 live poultry outbreak, it appears that PFGE is more discriminatory than CRISPR-MVLST, as CRISPR-MVLST also identified two non-outbreak related isolates as TST42. Given the available epidemiological data available, these two isolates do not appear to be associated with the outbreak. The fact that CRISPR-MVLST works better in some instances than others is not surprising and can also occur when other subtyping methods are used. ‘Problematic’ PFGE pulsotypes also exist and is one reason that second generation methods like MLVA and CRISPR-MVLST are being developed [33,52]. As a recent example, isolates associated with the 2012 *S.* Typhimurium cantaloupe outbreak, had a common PFGE pattern so additional subtyping by MLVA was performed to correctly define the outbreak strain [24]. That there is a strong association among closely related sequence types and closely related PFGE patterns for both *S.* Typhimurium (Figure 5) and *S.* Newport [41] provides further evidence that CRISPR-MVLST could serve as an appropriate alternative subtyping method.

Beyond the data shown here and in further evaluating the value of CRISPR-MVLST sequence typing, a recent study investigating *S.* Typhimurium isolates from a variety of animal sources showed an association of CRISPR-MVLST sequence types and resistance to antibiotics [40]. As part of that study, the most frequent TSTs were TST10 and TST42, both of which were found in this current study. TST10 was also the most frequent clinical sequence type seen in this study (16/86 isolates) but only two isolates were TST42.

## Conclusion

CRISPR-MVLST is a relatively new subtyping approach with limited studies conducted in *Salmonella* that demonstrate its utility [33,34,39]. Our data here add to this body of work by demonstrating its functionality in two highly prevalent clinical serovars. Investigation of several more outbreak strains using CRISPR-MVLST will elucidate the true capability of this subtyping method. Our data here show that CRISPR-MVLST can be used in concert with PFGE, as in the case of *S.* Heidelberg, or potentially as an independent subtyping method, as in the case of *S.* Typhimurium.

## Methods

### Bacterial isolates and sample preparation

A summary of all isolates analyzed in this study is listed in Table 5. A total of 89 and 86 clinical isolates of *S.* Heidelberg and *S.* Typhimurium, respectively, were obtained from the Pennsylvania Department of Health. These isolates were selected systematically (isolates received closest to the 1^st^ and 15^th^ of each month from 2005 – 2011 were selected) to represent an unbiased collection of human clinical isolates. PFGE-*XbaI* analysis of these isolates was conducted using standard protocols [7,53]. All isolates were stored at -80°C in 20% glycerol. Isolates were grown overnight in 2 mL LB at 37°C in a shaking incubator. DNA was isolated using the Promega genomic DNA isolation kit, following the manufacturer’s directions (Promega, Madison, WI). DNA samples were stored at -20°C prior to PCR analysis.

### PCR amplification

Primers for amplification of all four genomic loci are listed in Table 6. PCR reactions were performed in a total volume of 25 μl: 1.5 μl template, 0.3 μl Taq (1.5 units; New England Bio Labs, Ipswich, MA), 0.2 μl 10 mM dNTPs, 1 μl of each 10 μM primer, 2.5 μl of 10× *Taq* buffer and 18.5 μl water. PCR conditions were as follows and the annealing temperatures (AT) are listed in Table 6: initial denaturation step of 10 minutes at 94°C followed by 35 cycles of 1 minute at 94°C, 1 minute at AT and extension for 1 minute (*fimH* and *sseL*) or 1.5 minutes (CRISPR1 and CRISPR2) at 72°C; a final extension step was done at 72°C for 8 minutes. 5 μl of each PCR product was electrophoretically analyzed on a 1.2% agarose gel and the remaining reaction stored at -20°C.

**Table 6:** List of primers used in this study for PCR amplification and sequencing of the four CRISPR-MVLST markers

**Primer**	**Orientation**	**Primer sequence (5′-3′)**	**Annealing temp.**	**PCR**	**Sequencing**
CRISPR1-5	Forward	TGAAAACAGACGTATTCCGGTAGATT	55.5	✓	✓
CRISPR1-1	Reverse	CAGCATATTGACAAGGCGCT		✓	✓
CRISPR2-3	Forward	ATTGTTGCGATTATGTTGGT	57	✓	✓
CRISPR2-1	Reverse	TCCAGCTCCCTTATGATTTT		✓	
CRISPR2-4	Reverse	GCAATACCCTGATCCTTAACGCCA			✓
CRISPR2-5	Reverse	CGACGAAATTAAAACCGAACT			✓
CRISPR2-6	Forward	CGGATTCCATGCGTTTTCA			✓
CRISPR2-7	Forward	CCGGCGAGGTCAATAAAA			✓
CRISPR2-8	Forward	TGACGCTGGTCTATACCG			✓
CRISPR2-9	Forward	GTGACGTCAGTGCCGAA			✓
CRISPR2-10	Reverse	CTCTTCGCACTCTCGATCAA			✓
fimH-1	Forward	AGGTGAACTGTTCATCCAGTGG	56.7	✓	✓
fimH-2	Reverse	GCGGGCTGAACAAAACACAA		✓	✓
sseL-1	Forward	AAAATCAGGTCTATGCCTGATTTAATATATC	60	✓	
sseL-2	Reverse	GGCTCTAAGTACTCACCATTACT		✓	
sseL-3	Forward	ACCAGGAAACAGAGCAAAATGAATATATGT			✓
sseL-4	Forward	TTCTCTCGGTAAACTATCCTATTGGGC			✓

### DNA sequencing

PCR products were treated with 10 units of Exonuclease (New England Bio Labs, Ipswich, MA) and 1 unit of Antarctic alkaline phosphatase (New England Bio Labs, Ipswich, MA). The mixture was incubated for 40 minutes at 37°C to remove remaining primers and unincorporated dNTPs. The enzymes were inactivated by incubating the samples at 85°C for 15 minutes. Purified PCR products were sequenced at the Huck Institute’s Nucleic Acid Facility at The Pennsylvania State University using 3’ BigDye-labeled dideoxynucleotide triphosphates (v 3.1 dye terminators; LifeTechnoloties, Carlsbad, CA) and run on an ABI 3730XL DNA Analyzer, using ABI Data Collection Program (v 2.0). Data was analyzed with ABI Sequencing Analysis software (Version 5.1.1). The primers used for sequencing are listed in Table 6. In total, four PCR reactions and eight sequencing reactions were conducted for each isolate being typed. Additionally, one internal sequencing reaction was required for 14/26 *S*. Typhimurium CRISPR2 alleles, due to the increased length of this locus. There were two alleles (only representing 2/86 *S.* Typhimurium isolates), 181 and 205, which required extra primers due to the presence of a duplicated region of the locus. The positions of these extra primers are shown in Additional file 1: Figure S1. CRISPR2 alleles that were sequenced using more than two primers are indicated in Table 3.

### Sequence analysis and sequence type assignment

Sequences were assembled and aligned using SeqMan and MegAlign, respectively (Lasergene 10, DNA Star, Madison, WI) and unique alleles were assigned a unique numerical designation. All sequences from this study were submitted as a batch to NCBI and the accession numbers (KF465853 - KF465929) are shown for each allele in Additional file 2. For each isolate the combination of allelic types at all four loci defines the serovar-designated sequence type (ST) (Tables 2 and 3), with each unique allelic type assigned a different ST number. The presence of a SNP in any marker was sufficient to define a new allele. Analysis of CRISPR1 and CRISPR2 was performed using CRISPR-finder (http://crispr.u-psud.fr/Server/). We did not identify any SNPs within either CRISPR locus that defined any allele. Allelic differences occurred from deletion of one or more spacers, addition of a spacer or duplication/triplication of a spacer. Discriminatory power was calculated using the method described by Hunter and Gaston [54], with strains defined as either unique STs or unique PFGE patterns.

Relationships between TSTs were calculated using BURST (http://www.pubmlst.org/analysis/), with a group definition of n-1*.* Unique PFGE patterns, or pulsotypes, were defined by PulseNet, using the Dice coefficient with an optimization of 1.5% and a position tolerance of 1.5%. The difference of one band is sufficient to call two PFGE patterns different. PFGE dendrograms were generated using BioNumerics v. 6.6.

### *S.* Typhimurium outbreak study

A summary of 30 *S.* Typhimurium outbreak isolates that were obtained from the Pennsylvania Department of Health is listed in Table 4. Ten of these isolates associated with an outbreak in 2004 (cluster 0411PAJPX-1c) where affected patients had been on a bus trip together, though no vector was ever identified. Another 10 isolates were linked to an outbreak in 2009 (cluster 0905PAJPX-1), which was associated with live poultry. The remaining 10 isolates represent sporadic case isolates, also from 2009 but were not associated with the 0905PAJPX-1 outbreak and thus served as controls. The isolates were cultured as described above.

### Consent and institutional review board (IRB) approval

This study design was reviewed by the Pennsylvania Department of Health IRB and was determined to be exempt under federal regulations as it falls within the category “research that involves the collection or study of existing data, documents, records, pathological specimens, or diagnostic specimens where the information is recorded by the investigator in such a manner that subjects cannot be identified, directly or through identifiers linked to the subjects”.

## Competing interests

The authors declare that they have no competing interests.

## Authors’ contributions

NS designed, coordinated and carried out the experiments and bioinformatics analyses and wrote the manuscript. CS isolated bacterial cultures and did the PFGE. MD and RB participated in the CRISPR alignment analysis. ED conceived of the study, participated in the design and coordination of the study and helped to write the manuscript. All authors read and approved the final manuscript.

## Supplementary Material

Additional file 1 **Location of CRISPR2 primers used for PCR and sequencing.** Representation of CRISPR2 spacers from three alleles (allele numbers shown on the left) with each unique spacer shown as a uniquely colored box. Regions of spacer duplication are indicated above the array with a black line. Allele 164 is the most frequent allele. Alleles 181 and 205 each only occurred in one isolate and given the length and the seven spacers that are duplicated (line 2), required five additional primers for sequencing. These were the only two isolates that required this many primers. The primers are indicated below the array. The PCR primers are shown in bold. With the exception of CR2-4, all were used for PCR and sequencing. Click here for file

Additional file 2 Accession Numbers Table listing the accession numbers for all alleles identified in this study. Click here for file
