# Supplementary material for: CRISPR-MVLST subtyping of Salmonella enterica subsp. enterica serovars Typhimurium and Heidelberg and application in identifying outbreak isolates
Source: BMC Microbiol. 2013 Nov 12;13:254. doi: 10.1186/1471-2180-13-254 (PMC3840669; doi:10.1186/1471-2180-13-254)
Supplement: Additional file 1 — Location of CRISPR2 primers used for PCR and sequencing. Representation of CRISPR2 spacers from three alleles (allele numbers shown on the left) with each unique spacer shown as a uniquely colored box. Regions of spacer duplication are indicated above the array with a black line. Allele 164 is the most frequent allele. Alleles 181 and 205 each only occurred in one isolate and given the length and the seven spacers that are duplicated (line 2), required five additional primers for sequencing. These were the only two isolates that required this many primers. The primers are indicated below the array. The PCR primers are shown in bold. With the exception of CR2-4, all were used for PCR and sequencing. [file 1471-2180-13-254-S1.pdf]

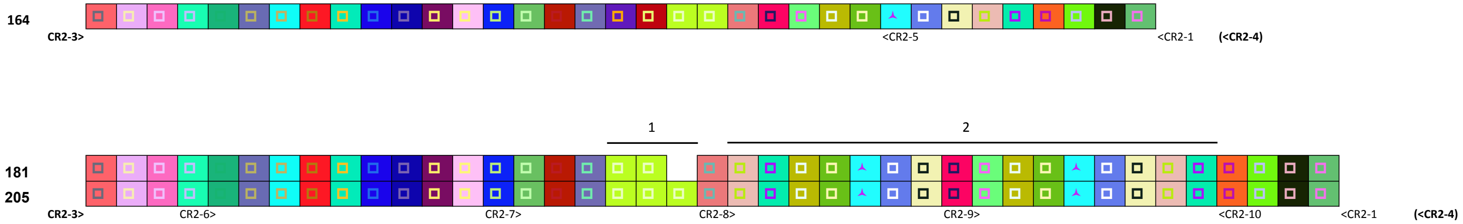

Supplemental Figure 1. Location of CRISPR2 primers used for PCR and sequencing

Representation of CRISPR2 spacers from three alleles (allele numbers shown on the left) with each unique spacer shown as a uniquely colored box. Regions of spacer duplication are indicated above the array with a black line. Allele 164 is the most frequent allele. Alleles 181 and 205 each only occurred in one isolate and given the length and the seven spacers that are duplicated (line 2), required five additional primers for sequencing. These were the only two isolates that required this many primers. The primers are indicated below the array. The PCR primers are shown in bold. With the expection of CR2-4, all were used for PCR and sequencing.
