# Supplementary material for: CRISPR-MVLST subtyping of Salmonella enterica subsp. enterica serovars Typhimurium and Heidelberg and application in identifying outbreak isolates
Source: BMC Microbiol. 2013 Nov 12;13:254. doi: 10.1186/1471-2180-13-254 (PMC3840669; doi:10.1186/1471-2180-13-254)
Supplement: Additional file 2 — Accession Numbers Table listing the accession numbers for all alleles identified in this study. [file 1471-2180-13-254-S2.doc]

Additional File 2.

| **Gene** | **Allele** | **Accesion Number** |
| --- | --- | --- |
| *fimH* | 6 | KF465855 |
|  | 8 | KF465856 |
|  | 17 | KF465853 |
|  | 18 | KF465854 |
|  | 49 | KF465857 |
|  | 50 | KF465858 |
| *sseL* | 15 | KF465861 |
|  | 19 | KF465859 |
|  | 20 | KF465862 |
|  | 61 | KF465863 |
|  | 62 | KF465864 |
|  | 63 | KF465865 |
|  | 67 | KF465860 |
| CRISPR1 | 10 | KF465879 |
|  | 11 | KF465880 |
|  | 129 | KF465881 |
|  | 130 | KF465882 |
|  | 131 | KF465883 |
|  | 132 | KF465884 |
|  | 133 | KF465885 |
|  | 134 | KF465886 |
|  | 135 | KF465887 |
|  | 136 | KF465888 |
|  | 137 | KF465889 |
|  | 138 | KF465890 |
|  | 139 | KF465891 |
|  | 140 | KF465892 |
|  | 141 | KF465893 |
|  | 142 | KF465894 |
|  | 143 | KF465895 |
|  | 166 | KF465896 |
|  | 167 | KF465866 |
|  | 168 | KF465867 |
|  | 169 | KF465868 |
|  | 170 | KF465869 |
|  | 171 | KF465870 |
|  | 172 | KF465871 |
|  | 173 | KF465872 |
|  | 174 | KF465873 |
|  | 175 | KF465874 |
|  | 176 | KF465875 |
|  | 177 | KF465876 |
|  | 178 | KF465877 |
|  | 179 | KF465878 |
| CRISPR2 | 32 | KF465897 |
|  | 159 | KF465905 |
|  | 160 | KF465906 |
|  | 161 | KF465907 |
|  | 162 | KF465908 |
|  | 163 | KF465909 |
|  | 164 | KF465910 |
|  | 165 | KF465911 |
|  | 167 | KF465912 |
|  | 168 | KF465913 |
|  | 169 | KF465914 |
|  | 170 | KF465915 |
|  | 171 | KF465916 |
|  | 172 | KF465917 |
|  | 173 | KF465918 |
|  | 174 | KF465919 |
|  | 175 | KF465920 |
|  | 177 | KF465921 |
|  | 178 | KF465922 |
|  | 179 | KF465923 |
|  | 180 | KF465924 |
|  | 181 | KF465926 |
|  | 205 | KF465925 |
|  | 207 | KF465928 |
|  | 208 | KF465929 |
|  | 209 | KF465898 |
|  | 210 | KF465899 |
|  | 211 | KF465900 |
|  | 212 | KF465901 |
|  | 213 | KF465902 |
|  | 214 | KF465903 |
|  | 215 | KF465904 |
